# Correction: Sociodemographic characteristics and risk factors related to SARS-CoV-2 infection in Luanda, Angola

**DOI:** 10.1371/journal.pone.0252254

**Published:** 2021-05-20

**Authors:** Cruz S. Sebastião, Zoraima Neto, Pedro Martinez, Domingos Jandondo, Janete Antonio, Manuela Galangue, Marcia de Carvalho, Kumbelembe David, Julio Miranda, Pedro Afonso, Luzia Inglês, Raisa Rivas Carrelero, Jocelyne Neto de Vasconcelos, Joana Morais

There are errors in the author affiliations. The correct affiliations are as follows:

Cruz S. Sebastião^1,2,3^, Zoraima Neto^1^, Pedro Martinez^1^, Domingos Jandondo^1^, Janete Antonio^1^, Manuela Galangue^1^, Marcia de Carvalho^1^, Kumbelembe David^1^, Julio Miranda^1^, Pedro Afonso^1^, Luzia Inglês^1^, Raisa Rivas Carrelero^1^, Jocelyne Neto de Vasconcelos^1,2^, Joana Morais^1,4^

**1** Instituto Nacional de Investigação em Saúde, Luanda, Angola, **2** Centro de Investigação em Saúde de Angola, Caxito, Angola, **3** Instituto Superior de Ciências da Saúde, Universidade Agostinho Neto, Luanda, Angola, **4** Faculdade de Medicina, Universidade Agostinho Neto, Luanda, Angola.
